# Inadequate Riboflavin Intake and Anemia Risk in a Chinese Population: Five-Year Follow Up of the Jiangsu Nutrition Study

**DOI:** 10.1371/journal.pone.0088862

**Published:** 2014-02-12

**Authors:** Zumin Shi, Shiqi Zhen, Gary A. Wittert, Baojun Yuan, Hui Zuo, Anne W. Taylor

**Affiliations:** 1 Department of Nutrition and Foodborne Disease Prevention, Jiangsu Provincial Centre for Disease Control and Prevention, Nanjing, China; 2 Discipline of Medicine, University of Adelaide, Adelaide, Australia; Kyushu University Faculty of Medical Science, Japan

## Abstract

**Objectives:**

Riboflavin (vitamin B2) has been shown in animal studies to affect the absorption and metabolism of iron. Cross-sectional population studies show a relationship between riboflavin intake and anemia but prospective population studies are limited. The aim of the study was to determine the relationship between riboflavin intake and the risk of anemia in a Chinese cohort.

**Method:**

The study used data from 1253 Chinese men and women who participated in two waves of the Jiangsu Nutrition Study (JIN), five years apart, in 2002 and 2007. Riboflavin intake and hemoglobin (Hb) were quantitatively assessed together with dietary patterns, lifestyle, socio-demographic and health-related factors.

**Results:**

At baseline, 97.2% of participants had inadequate riboflavin intake (below the estimate average requirement). Riboflavin intake was positively associated with anemia at baseline, but low riboflavin intake was associated with an increased risk of anemia at follow-up among those anemic at baseline. In the multivariate model, adjusting for demographic and lifestyle factors and dietary patterns, the relative risk and 95% confidence interval for anemia at follow-up, across quartiles of riboflavin intake were: 1, 0.82(0.54–1.23), 0.56(0.34–0.93), 0.52(0.28–0.98) (p for trend 0.021). There was a significant interaction between riboflavin and iron intake; when riboflavin intake was low, a high iron intake was associated with a lower probability of anemia at follow-up. This association disappeared when riboflavin intake was high.

**Conclusion:**

Inadequate riboflavin intake is common and increases the risk of anemia in Chinese adults. Given the interaction with iron intake correcting inadequate riboflavin intake may be a priority in the prevention of anemia, and population based measurement and intervention trials are required.

## Introduction

Despite the nutrition transition and improved nutritional status in many developing countries, anemia remains a major public health problem. In China, according to data from a national nutrition survey, more than 15% of the population were anemic in 2002, [1]. While iron deficiency is the main cause of anemia in developing countries [2], other nutritional factors, for example vitamins (vitamin A, vitamin C and vitamin B2) [3], are also important but have received considerably less attention. Riboflavin (vitamin B2) deficiency is one of the most common vitamin deficiencies in developing countries especially those with rice as the staple food coupled with insufficient milk and meat intake [4]. In animal studies, riboflavin has been shown to increase iron absorption [3], and riboflavin deficiency can significantly increase the rate of gastrointestinal iron loss as well as decrease the mobilization of iron from stores [5]. In humans riboflavin deficiency has been shown to negatively affect iron utilization [6].

In China the mean intake of riboflavin is around 0.8 mg/d, which is below the estimated average requirement (EAR, 1.4 mg/d for men aged 18 and above, 1.2 mg/d for women aged 18–49 years, 1.4 mg/d for women aged 50 years and above) [7]. In contrast, the iron intake in the population is over 23 mg/d [8], which is above the RNI (15 mg/d for men aged 18–49 years, 20 mg/d for women aged 18–49 years) [7].

The association between riboflavin intake and anemia in China has been inconsistent in cross-sectional studies. One case-control study in South-west China found that as compared to non-anemic elderly women, anemic elderly women had lower riboflavin intake [9]. Another Chinese study showed that there was no significant difference in riboflavin intake between anemic and non-anemic pregnant women [10]. The relationship between riboflavin intake and anemia has not, as far as we can determine, been prospectively investigated in a representative cohort study in China, or indeed elsewhere. The aim of the current study was to assess the association between riboflavin intake and the risk of anemia using the five year longitudinal data from the Jiangsu Nutrition Study. The second aim of the study was to assess the interaction between riboflavin intake and iron intake in relation to anemia.

## Subjects and Methods

### Study design

The methodologies of the Jiangsu Nutrition (JIN) Cohort Study have been described previously [11]–[13]. The baseline of the cohort was based on a subsample of the Chinese National Nutrition and Health Survey representing Jiangsu province in 2002. The rural sample was selected from six counties (Jiangyin, Taichang, Shuining, Jurong, Sihong and Haimen). From each of the six counties, three smaller towns were randomly selected. The urban sample was selected from the capital cities of the two prefectures, Nanjing and Xuzhou; and from each capital city three streets were randomly selected. In each town/street, two villages/neighbourhoods were randomly selected, and 90 households were further selected randomly from each village/neighbourhood. All the members in the households were invited to take part in the study. In addition, one third of the households were interviewed on dietary intake.

**Figure 1:**
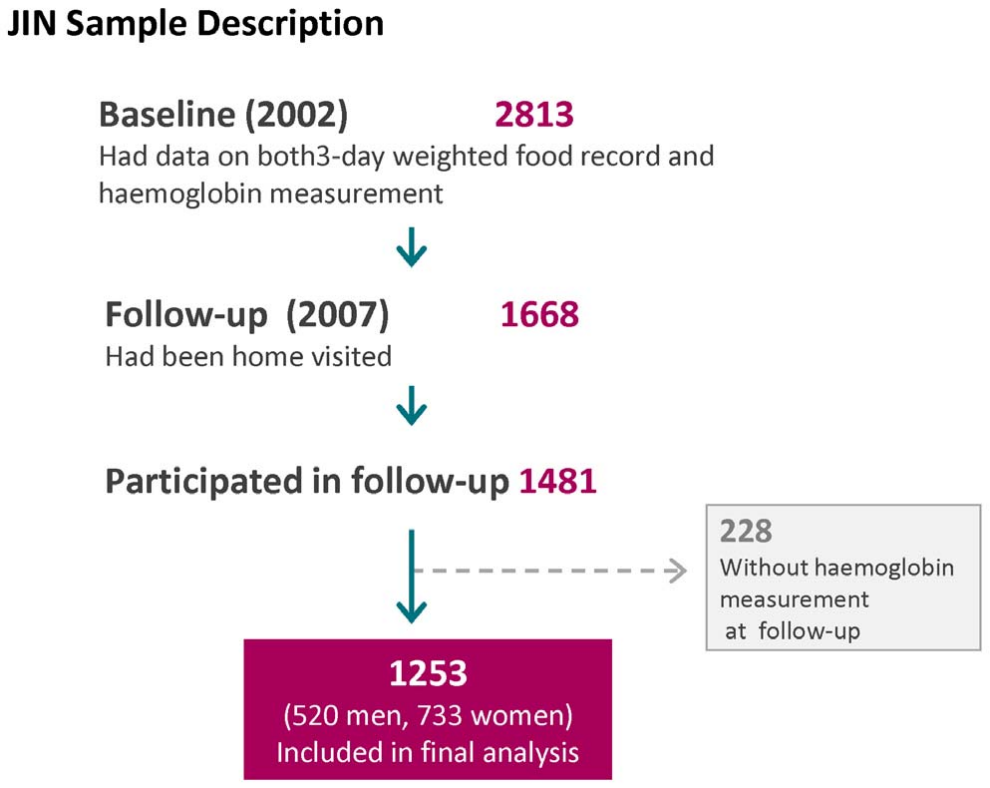
Sample description.

At baseline, 2813 adults aged 20 and above had detailed dietary information and blood hemoglobin measurement. At follow-up in 2007, 1668 participants were identified through household visits, and 1481 of them participated in the follow-up interview. After excluding 228 participants without hemoglobin values at follow-up, the final sample size examined for anemia status change consisted of 520 men and 733 women (total n = 1253) (Figure 1). The study was conducted according to the guidelines in the Declaration of Helsinki and all procedures involving human subjects/patients were approved by Jiangsu Provincial Centre for Disease Control and Prevention. Written consent to participate was obtained from all the participants.

### Data collection and measurements

Participants were interviewed at their homes by intensively trained health workers using a standard questionnaire [11].

#### Outcome variables

An overnight fasting blood sample (capillary and venous) was collected at baseline and follow-up. The capillary blood samples were analyzed for hemoglobin (Hb) by the cyanmethemoglobin method [14] in the local Centers for Disease Control and Prevention. The venous blood plasma was separated by centrifugation at 3,200 rpm for 10–15 min within 1 h of collection, kept at room temperature without sunshine and sent to central laboratory for biochemical tests. Serum ferritin was analyzed only at baseline in a laboratory in the National Centre for Disease Control and Prevention in Beijing using a commercially available radioimmunoassay kit (Beijing North Institute of Biological Technology). Anemia was defined as a Hb level below 13 g/dL for men and 12 g/dL for women [15]. Iron deficiency anemia (IDA) was defined as the presence of both anemia and a serum ferritin level <15 µg/l.

#### Dietary intake

In 2002 and 2007, dietary intake patterns during the previous year were investigated by a series of detailed questions about the usual frequency and quantity of intake of 33 food groups and beverages using a food frequency questionnaire (FFQ) administered by a trained health care worker. The FFQ administered in this manner has been validated with weighted food records [16]–[18]. Baseline riboflavin intake (*exposure variable*) and other nutrients (e.g. vitamin C, protein), alcohol and vegetable oil intakes were assessed using a 3-day weighed food diary which recorded all foods consumed by each individual, on three consecutive days (including one weekend); this 3-day weighed food diary was not undertaken at follow-up due to its high cost and time needed. At the beginning and end of the 3-day survey, health workers weighed all the food stocked in the household. Each day, all purchases, home production, and processed snack foods were weighed and recorded. Food intakes of each individual in the household were recorded in detail each day.

During the interview, the health workers would check any intake value for a particular food that fell below or above the usual intake value by the population in the region. Food consumption data were analyzed using the Chinese Food Composition Table [19]. Inadequate riboflavin intake was defined as the usual riboflavin intake below the Chinese EAR (1.4 mg/d for men aged 18 and above, 1.2 mg/d for women aged 18–49 years, 1.4 mg/d for women aged 50 years and above) [7]. To estimate the prevalence of inadequate riboflavin intake, we used the EAR cut point method [20]. We used the National Cancer Institute (NCI) method to estimate the distribution of usual riboflavin intake [21].

#### Dietary patterns

Baseline dietary patterns were identified by factor analysis based on food intake estimated by the FFQ, using standard principal component analysis as described elsewhere [22]. Four food patterns were obtained: Factor 1 (‘macho’) included various kinds of animal foods and alcohol; Factor 2 (the ‘traditional’ pattern) loaded heavily on rice, fresh vegetables and inversely on wheat flour; Factor 3 (‘sweet tooth’) contained cake, milk, yoghurt and drinks; and, Factor 4 (‘vegetable rich’ pattern) was characterized whole grains, fruits, root vegetables, fresh and pickled vegetables, milk, eggs and fish. The four factors explained 28.5% of the variance in intake. Similarly four dietary patterns were identified at follow up. The correlation coefficient for corresponding dietary patterns between baseline and follow up ranged from 0.139 (‘vegetable rich’ pattern), 0.241(‘macho’ pattern, p<0.001), 0.254(‘sweet tooth’ pattern, p<0.001) to 0.593 (‘traditional’ pattern, p<0.001).

#### Covariates

Cigarette smoking was assessed by asking the frequency of daily cigarette smoking in the past 30 days. Education was recoded into either ‘Low’ (illiteracy, primary school); ‘Medium’ (junior middle school); or, ‘High’ (high middle school or higher), based on six categories of education levels in the questionnaire. Occupation was recoded into ‘Manual’ or ‘Non-manual’ based on a question with 12 occupational categories. Hypertension medication use (yes/no) was asked at baseline and follow-up.

In both baseline and follow-up, anthropometric measurements were obtained using standard protocols and techniques [18], [23], including body weight, height, and blood pressure. Body weight was measured in light indoor clothing to the nearest 100 grams, and height to the nearest mm. Using the Chinese classification, BMI is categorized as normal (BMI<24 kg/m^2^), overweight (BMI 24–27.9 kg/m^2^), and obese (BMI≥28 kg/m^2^)[24]. Blood pressure was measured twice by mercury sphygmomanometer on the right upper arm of the subject, who was seated for 5 min before the measurement. The mean of these two measurements was used in the analyses. The cuff size was selected on the basis of the upper arm circumference to ensure that the cuff did not overlap [23]. Hypertension was defined as systolic blood pressure above 140 mmHg and/or diastolic blood pressure above 90 mmHg, or use of antihypertensive medications.

### Statistics

Riboflavin intake was coded into quartiles. Chi square test was used to compare differences between categorical variables, and ANOVA was used to compare differences in continuous variables between groups. Poisson regression with robust variance models were also used to assess the association between riboflavin intake quartiles and anemia at follow up [25]. Prevalence rate ratio was calculated and adjusted for age, sex, energy intake, iron intake (model 1), and further adjustment for education, occupation, smoking, hypertension, overweight (yes/no) at baseline, energy, iron and vitamin C intake (as continuous variables), baseline dietary patterns (model 2), and follow up dietary patterns (model 3). Tests of linear trends across riboflavin quartiles were computed using ordinal scoring. Food patterns were also put into the multivariate models to control for residual confounding, as suggested by Imamura *et al* [26]. To assess the interaction between riboflavin intake and iron intake, we treated both variables as continuous and put the product term of the variables in a logistic regression adjusting for potential confounding variables (variables adjusted in above model 2). After the logistic regression, we used the margins command to determine the adjusted probability (marginal effect) of anemia at follow up according to riboflavin intake and iron intake at baseline. For graphical visualization of the predicted probability of anemia at follow up, we used the “marginsplot” command, and the median of each quartile of iron intake was used. In the provinces, a distinct definition of urban/rural dwelling is difficult due to ongoing economic development. Since we have adjusted for education and job status as well as dietary patterns, the decision was made not to adjust for urban/rural. All the analyses were performed using STATA 12 (Stata Corporation, College Station). Statistical significance was considered to be when p<0.05 (two sided).

## Results

At baseline, among 1253 participants, there were 124 (23.9%) men and 261 (35.6%) women with anemia. The prevalence of iron deficiency anemia at baseline was 3.8% (0.8% in men, 5.9% in women). Of the 1253 participants followed up for five years, 13.1% developed anemia, while anemia resolved in 21.5% for the total sample. Only 9.3% of the participants had persistent anemia at the 5-year follow-up.

Individuals lost to follow up were younger (45.2 vs 49.4 years), were more likely to have high education (20.8% vs 10.9%, p<0.001), had higher Hb (13.6 vs 13.2 g/dl, p<0.001) and a lower prevalence of anemia (21.5% vs 30.7%, p<0.001), but there were no differences in mean BMI (p = 0.324), or energy (p = 0.246) and riboflavin intake (p = 0.358).

The mean intake of riboflavin at baseline was 0.80 (SD 0.33) mg/d. Overall 97.2% (98.0% women and 96.2% men) of the sample had usual riboflavin intake below Chinese EAR. The mean intake of iron was 24.9 mg/d (26.9 mg/d in men, 23.4 mg/d in women). Income and education were positively associated with riboflavin intake (**Table 1**). There were significant differences in energy, fat, protein, iron, vitamin C, and fiber intakes across quartiles of riboflavin intake. Riboflavin intake was positively associated with ferritin and association remained after adjusting for age and gender (data not shown).

**Table 1:** Baseline sample characteristics according to quartiles of riboflavin intake (n = 1253). ^a^

	Q1	Q2	Q3	Q4	p^b^
	(n = 313)	(n = 313)	(n = 313)	(n = 314)	
Age	51.3(14.0)	49.9(13.0)	48.9(12.7)	47.4(12.6)	0.002
BMI (kg/m^2^)	23.3(3.7)	23.5(3.5)	23.6(3.4)	23.3(2.9)	0.671
Overweight/obesity (%) (BMI≥24 kg/m^2^)	38.2	40.3	44.4	37.8	0.314
Hemoglobin (g/dL)	13.0(1.7)	13.0(1.7)	13.3(1.7)	13.5(1.9)	<0.001
Ferritin (µg/l)	81.1(66)	93.4(78.7)	104.3(81.8)	111.1(89.7)	<0.001
Ferritin <15 µg/l (%)	10.0	8.7	8.4	6.8	0.001
Nutrient intake					
Energy (kcal)	1841.9(460.4)	2210.5(458.6)	2512.8(575.2)	2766.2(677.7)	<0.001
Fat (g/d)	59.8(24.5)	74.4(28.2)	90.3(35.2)	101.7(39.6)	<0.001
Protein (g/d)	53(12.2)	66.5(12.7)	78.1(15.4)	92.1(23.3)	<0.001
Total iron (mg/d)	19.3(7.6)	21.9(6.2)	25.9(7.3)	32.5(11.1)	<0.001
Non-heme iron (mg/d)	18.2(7.9)	20.1(6.5)	23.3(7.4)	27.8(11.5)	<0.001
Heme iron (mg/d)	1.1(1.2)	1.8(1.9)	2.6(3)	4.7(5.7)	<0.001
Vitamin C (mg/d)	45.3(22.9)	63.4(33.3)	66.5(33.5)	84.8(45.5)	<0.001
Riboflavin (mg/d)	0.5(0.1)	0.7(0)	0.8(0.1)	1.3(0.3)	<0.001
Fiber (g/d)	10.3(9.6)	10.5(7.9)	11.2(6.5)	14.4(12.1)	<0.001
Alcohol intake (g/d)	0.7(3.7)	1.4(4.9)	2(5.6)	3.1(6.1)	<0.001
Women (%)	77.7	63.9	49.5	42.8	<0.001
Anemia (baseline) (%)	30.9	32.6	31.0	28.4	0.730
Hypertension (%)	31.8	28.1	33.5	29.7	0.475
Smoker (%)	16.9	21.4	30.4	39.6	<0.001
Alcohol drinker (%)	12.4	18.2	30.0	38.3	<0.001
Education					
Low (%)	64.3	57.5	53.7	39.6	
Medium (%)	28.0	32.6	35.8	44.7	<0.001
High (%)	7.6	9.9	10.5	15.7	
Manual job (%)	51.6	48.6	53	50.2	0.710
Income					
Low (%)	34.7	27.3	19.6	18.8	
Medium (%)	33.4	38.6	36.9	25.2	<0.001
High (%)	31.8	34.1	43.6	56.0	

a Values are presented as mean(SD) or percentage.

b p values were generated by chi-squared test for categorical variables and ANOVA test for continuous exposures.

At baseline, there was a positive association between riboflavin intake and anemia in women but not in men (**Table 2**). Across quartiles of riboflavin intake, the prevalence rate ratio (model 2) was 1.15(0.69–1.90), 0.97(0.53–1.75), and 1.01(0.50–2.05) in men (p for trend 0.821); 1.13(0.87–1.48), 1.40(1.04–1.89), and 1.32(0.93–1.88) in women (p for trend 0.046). There was a significant interaction between age and riboflavin intake in women: the positive association between riboflavin intake and anemia was only significant in women below age of 50 years (p for interaction 0.029) (data not shown).

**Table 2:** Baseline association between riboflavin intake (quartiles) and anemia.^a^

	Riboflavin intake quartiles in 2002				*P* for trend
	Q1	Q2	Q3	Q4	
	(0.5 mg/d)^b^	(0.7 mg/d)	(0.8 mg/d)	(1.3 mg/d)	
All participants (n = 1253)					
Model 1^c^	1	1.20(0.94–1.52)	1.28(0.99–1.67)	1.36(1.02–1.83)	0.033
Model 2^d^	1	1.13(0.89–1.43)	1.19(0.91–1.56)	1.22(0.89–1.69)	0.201
Men (n = 520)					
Model 1^c^	1	1.23(0.73–2.04)	1.08(0.61–1.90)	1.34(0.73–2.45)	0.465
Model 2^d^	1	1.15(0.69–1.90)	0.97(0.53–1.75)	1.01(0.50–2.05)	0.821
Women (n = 733)					
Model 1^c^	1	1.17(0.90–1.53)	1.48(1.11–1.97)	1.50(1.08–2.09)	0.004
Model 2^d^	1	1.13(0.87–1.48)	1.40(1.04–1.89)	1.32(0.93–1.88)	0.046

a Values are prevalence rate ratio (95% CI) from Poisson regression.

b mean riboflavin intake with quartile.

c Model 1 adjusted for age (years, as continuous), sex (not adjusted in sex specific model), energy and iron intake.

d Model 2 adjusted for age, sex (not adjusted in sex specific model), smoking (0, 1–19, ≥20 cigarettes/day), alcohol drinking (g/day), education (low, medium, high), and occupation (manual/non-manual), overweight (BMI≥24 kg/m^2^, yes/no), hypertension (yes/no), intake of energy (kcal/day), iron (mg/day), and vitamin C (mg/day) (as continuous variables), for baseline dietary patterns.

The risk of anemia at follow-up in relation to riboflavin intake and anemic status at baseline is shown in Table 3. In the absence of anemia at baseline riboflavin intake had no association with anemia at follow-up. However, among those anemic at baseline, there was a clear inverse association between riboflavin intake and likelihood of anemia at follow-up. The relative risk and 95% confidence intervals for anemia at follow-up across quartiles of riboflavin intake were (model 3): 1, 0.82(0.54–1.23), 0.56(0.34–0.93), 0.52(0.28–0.98) (p for trend 0.021). Additional adjustment for other B vitamins and folate intake did not change the association between riboflavin intake and anemia (data not shown).

**Table 3:** Relative risk (95% CI) for anemia at follow-up derived from Poisson regression according to quartiles of baseline riboflavin intake among Chinese adults by anemic status at baseline in Jiangsu Nutrition Study.^a^

	Riboflavin intake quartiles in 2002				P for trend
	Q1	Q2	Q3	Q4	
	(0.5 mg/d)^b^	(0.7 mg/d)	(0.8 mg/d)	(1.3 mg/d)	
Non-anemic at baseline (n = 868)					
Model 1^c^	1	1.16(0.80–1.68)	0.83(0.53–1.31)	0.97(0.61–1.54)	0.571
Model 2^d^	1	1.17(0.81–1.68)	0.84(0.53–1.33)	1.00(0.59–1.68)	0.663
Model 3^e^	1	1.17(0.81–1.68)	0.80(0.50–1.28)	1.00(0.59–1.69)	0.621
Anemic at baseline (n = 385)					
Model 1^c^	1	0.89(0.60–1.32)	0.61(0.38–0.97)	0.61(0.37–1.00)	0.020
Model 2^d^	1	0.82(0.54–1.23)	0.57(0.35–0.93)	0.52(0.28–0.97)	0.017
Model 3^e^	1	0.82(0.54–1.23)	0.56(0.34–0.93)	0.52(0.28–0.98)	0.021

a Values are prevalence rate ratio (95% CI) from Poisson regression.

b mean riboflavin intake with quartile.

c Model 1 adjusted for age (years, as continuous), sex, and energy intake.

d Model 2 adjusted for age, sex, smoking (0, 1–19, ≥20 cigarettes/day), alcohol drinking (g/day), education (low, medium, high), and occupation (manual/non-manual), overweight (BMI≥24 kg/m^2^, yes/no), hypertension (yes/no), intake of energy (kcal/day), iron (mg/day), and vitamin C (mg/day) (as continuous variables), and baseline dietary patterns.

e Model 3 additional adjusted for dietary patterns (continuous) at follow-up.

**Figure 2:**
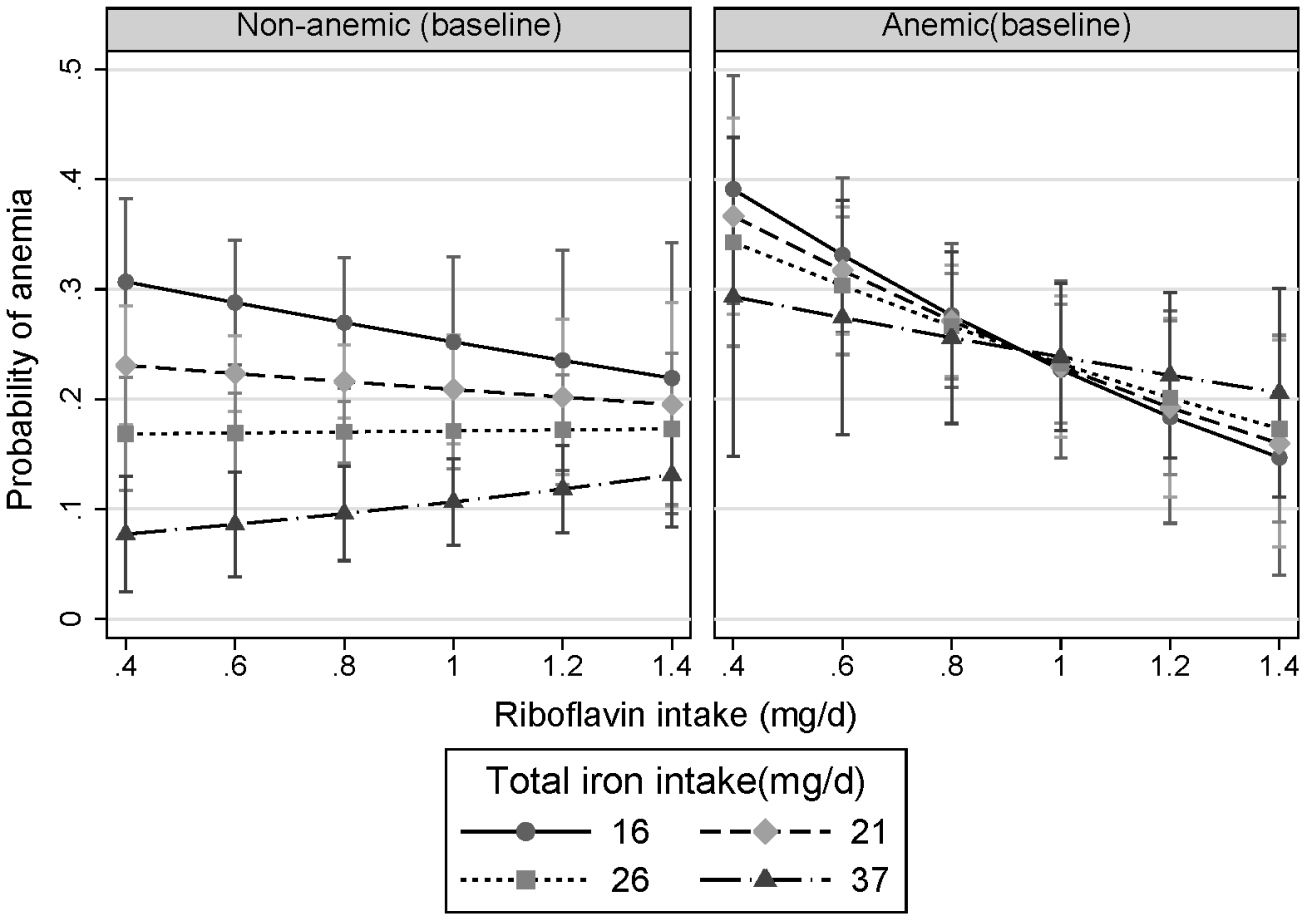
Interaction between riboflavin intake and iron intake at baseline in relation to anemia at follow up stratified by anemia status at baseline. Marginsplot syntax was used to make the plot after logistic regression adjusting for age, gender, smoking, energy intake, dietary patterns (continuous), education, income, BMI, and hypertension at baseline. The values represent the adjusted probability of anemia at follow-up. The values of iron intake represent the median intake in each quartile of iron intake. P value for interaction between riboflavin intake and iron intake was 0.008.

There was an interaction between riboflavin intake and iron intake at baseline according to the presence or absence of anemia (riboflavin and iron intake interaction, P = 0.008) (**Figure 2**). Among those non-anemic at baseline, when riboflavin intake was low, there was a strong inverse association between iron intake and the risk of anemia at follow up. However, the association between iron intake and anemia was weak when riboflavin intake was high. Among those anemic at baseline, a high riboflavin intake at baseline was associated with lower risk of persistent anemia, independent of iron intake.

**Figure 3:**
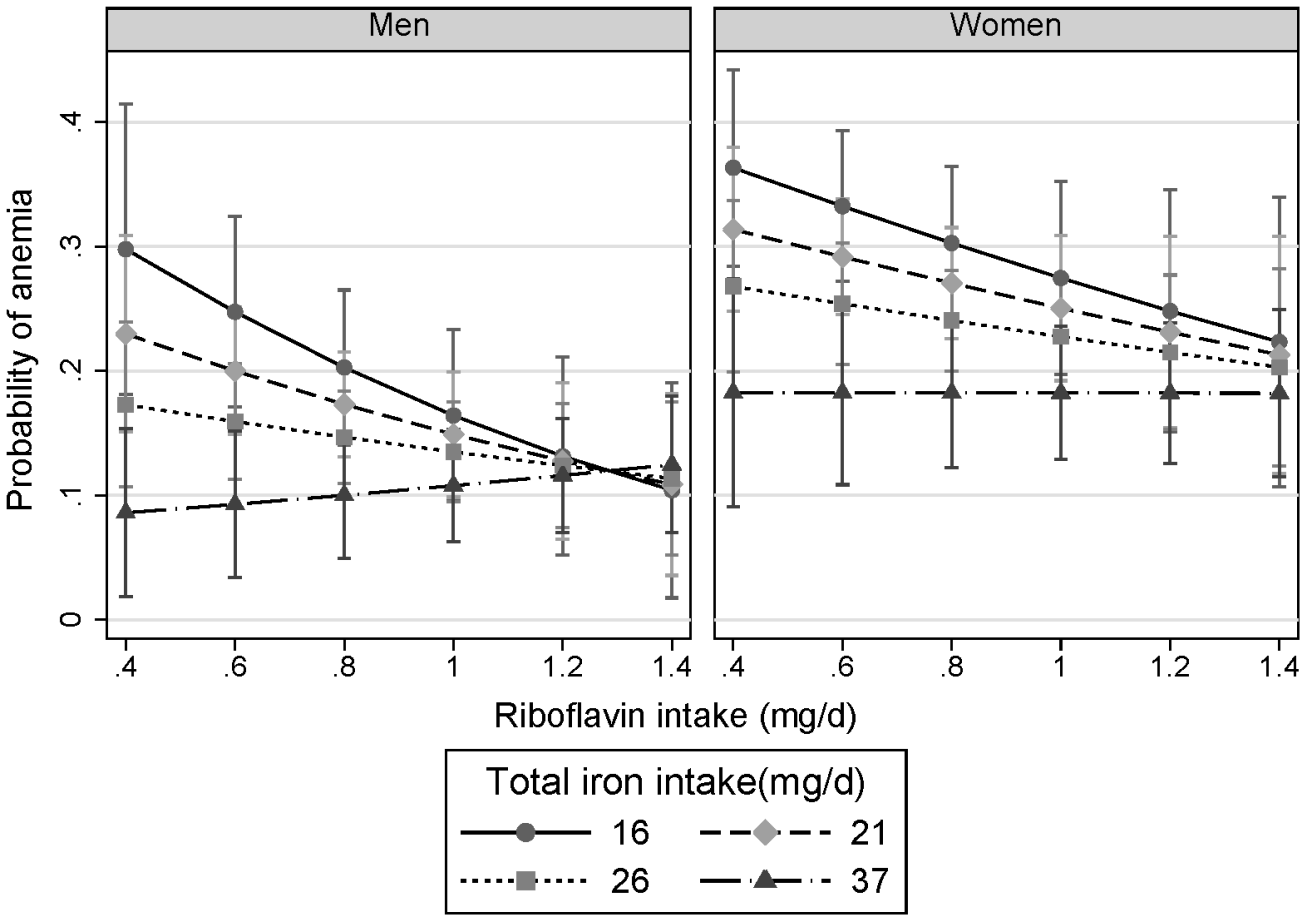
Interaction between riboflavin intake and iron intake at baseline in relation to anemia at follow up stratified by gender. Marginsplot syntax was used to make the plot after logistic regression adjusting for age, smoking, energy intake, dietary patterns, education, income, BMI, and hypertension at baseline. Anemia status at baseline was also adjusted in the model. The values of iron intake represent the median intake in each quartile of iron intake. The values represent the adjusted probability of anemia at follow-up. P for interaction between riboflavin and iron intake was 0.016.

The interaction between riboflavin and iron intake at baseline was similar in men and women (**Figure 3**). The predicted probability of anemia at follow up was 0.30(95%CI, 0.18–0.41) in men with riboflavin intake of 0.4 mg/d and iron intake of 16 mg/d. The corresponding figure was 0.12(0.07–0.16) for men with riboflavin intake of 1.2 mg/d and iron intake of 37 mg/d. When riboflavin intake reached 1.4 mg/d, iron intake was not related to the probability of anemia in the sample.

## Discussion

In this prospective study, we found that inadequate riboflavin intake was common, and associated with an increased risk of persistent anemia. There was a significant interaction between riboflavin intake and iron intake in relation to anemia risk.

Riboflavin deficiency, based on either intake of riboflavin or measurement of a serum biomarker (erythrocyte glutathione reductase activation coefficient, EGRAC) is common in many populations. For example, in Taiwan, it has been shown that about one in four elderly had marginal riboflavin deficiency based on riboflavin biomarker EGRAC [27]. Data from the China Nutrition and Health Survey (CHNS) showed that there was a persistent low intake of riboflavin in the Chinese population: the mean intake was around 0.7–0.9 mg/d among adults aged 18–45 years in six surveys between 1989 and 2004 [8]. This low intake of riboflavin may partly be due to the low consumption of milk. The mean intake of milk was only 2 g/d in 1989 and 12 g/d in 2004 [8]. In our study, only 6.5% of participants had an adequate intake of riboflavin.

There was a positive association between riboflavin intake and anemia among women, particularly in those below 50 years. In the same study population, we have previously reported that at baseline there was a positive association between total meat consumption and anemia: OR for anemia across quartiles of meat consumption were 1.00, 1.20, 1.39, 1.37(1.09–1.73), respectively [28]. Accordingly the apparent inconsistency may be the result of reverse causation in some of the study population. Women with anemia may have changed their diet and consequently increased riboflavin intake but had persistent anemia because of ongoing menstrual related blood loss.

In contrast to some cross sectional studies [10], [29], but consistent with a prior case control study [9], this longitudinal study shows that inadequate riboflavin intake is an important risk factor for anemia in China. The findings are in keeping with the known biological role of riboflavin in enhancing iron absorption and utilization [5], [30] (when riboflavin intake is high, the ability to mobilise iron from ferritin to and utilise it for the synthesis Hb will be high), and beneficial effects of riboflavin supplementation in the prevention of anemia in some [3], [31], but not all studies [3]. Three of 7 riboflavin supplementation trials undertaken before 1990 and reviewed in 2000 by Fishman *et al* showed beneficial effects [3]. However, these supplementation studies were mainly performed in developing countries and with short term (six weeks to three months) and small sample sizes (between 27 and 200). A recently published trial, undertaken in China, showed that retinol and riboflavin supplements decreased the prevalence of anemia in pregnant women also taking iron and folic acid supplements [32]. In addition a recent clinical trial in United Kingdom found that riboflavin supplementation can improve hematologic status among women aged 19–25 years with moderate riboflavin-deficiency [31].

The mean intake of iron (27 mg/d in men, 23 mg/d in women) exceeds the Chinese recommendation, suggesting there is adequate intake of iron. The majority of iron intake is in the form of non-heme iron. As the traditional Chinese diet is low in meat and milk and high in vegetable and other plant foods, the iron bioavailability is low [33]. The substantial decrease in prevalence of anemia in China over the past few decades may partly be due to the overall improvement of living condition and access to health service as well as the control of infectious diseases, and improvements in diet. The fact that the consumption of animal food has increased (75 g/d in 1989; 122 g/d in 2004) while the intake of cereals and starchy roots has declined [8], may increase in the bioavailability of iron in some degree. However, there was no substantial increase in the riboflavin intake over the past decades [8], probably due to the low intake of milk in the population. Despite the high prevalence of anemia in the sample, the level of ferritin is relatively normal. Among the anemic, only a small proportion of the participants had ferritin <15 µg/l. This may be due to the fact that when riboflavin is inadequate, the mobilization of iron from ferritin is limited [34].

The study raises questions about the current anemia prevention strategy of using iron fortified soy sauce, which started in 2004 after a substantial drop of the prevalence of anemia between 1982 and 2002. Iron supplementation may not be the best way to prevent anemia in the population when riboflavin intake is inadequate. In the short term, riboflavin supplement is required. Riboflavin alone without additional iron supplement has been shown to improve hematologic status in young women in the United Kingdom [31]. Riboflavin may have a role in the prevention of cancer and cardiovascular disease [35], and iron overload has been implicated in the etiology of type 2 diabetes mellitus [36], accordingly increasing dietary riboflavin may have significant overall public health benefits for the Chinese population where non communicable chronic disease is becoming epidemic.

A limitation of the study is the large number of individuals lost to follow up. Those lost to follow-up were younger (better educated and with lower prevalence of anemia) and as compared to the individuals who remained in the cohort they would have been at a lower risk of nutritionally-related anemia. The study included more women than men (possibly due to the fact that men in the rural area were more likely to migrate to urban area for work). This may limit the ability to generalize our findings to the general population. A further limitation is that absence of a biomarker for riboflavin status (e.g. EGRAC) and the use of ferritin levels to assess iron status in the absence of a marker of inflammation. We did not measure serum ferritin at follow-up. Finally, information on medical conditions (e.g. gastrointestinal bleeding) which may cause blood loss is not available. The strength of the study is its relatively large sample and detailed information on dietary intake at baseline. We were able to adjust for a range of confounding factors including dietary patterns at both time points.

In conclusion, riboflavin intake was largely inadequate in the Chinese population. Low intake of riboflavin is associated with increased risk of anemia. When riboflavin intake is adequate, there is no association between iron intake and anemia in the population. Correcting riboflavin deficiency may therefore be one of the components in the prevention of anemia, and population based measurement and intervention trials are required.
